# UltraCAST: A Flexible All-In-One Suicide Vector for Modifying Bacterial Genomes Using a CRISPR-Associated Transposon

**DOI:** 10.17912/micropub.biology.001721

**Published:** 2025-08-02

**Authors:** Anthony J. VanDieren, Jeffrey E. Barrick

**Affiliations:** 1 Department of Molecular Biosciences, The University of Texas at Austin

## Abstract

CRISPR-associated transposons (CASTs) are RNA-guided mobile genetic elements that are widespread in bacterial genomes. Here, we describe the UltraCAST, a suicide vector with the *Vibrio cholerae* Type I-F CAST system and Golden Gate assembly sites with fluorescent protein gene dropouts for guide RNA and a mini-transposon cargo cloning. We show an example of UltraCAST genome editing by disrupting a gene in the chromosome of *Serratia symbiotica* CWBI-2.3 ^T^ , a culturable relative of aphid endosymbionts. The UltraCAST can be used to flexibly insert DNA into specific genomic sites and facilitates testing this genome editing platform in non-model bacterial species that lack genetic tools.

**Figure 1. UltraCAST editing of the Serratia symbiotica genome:**
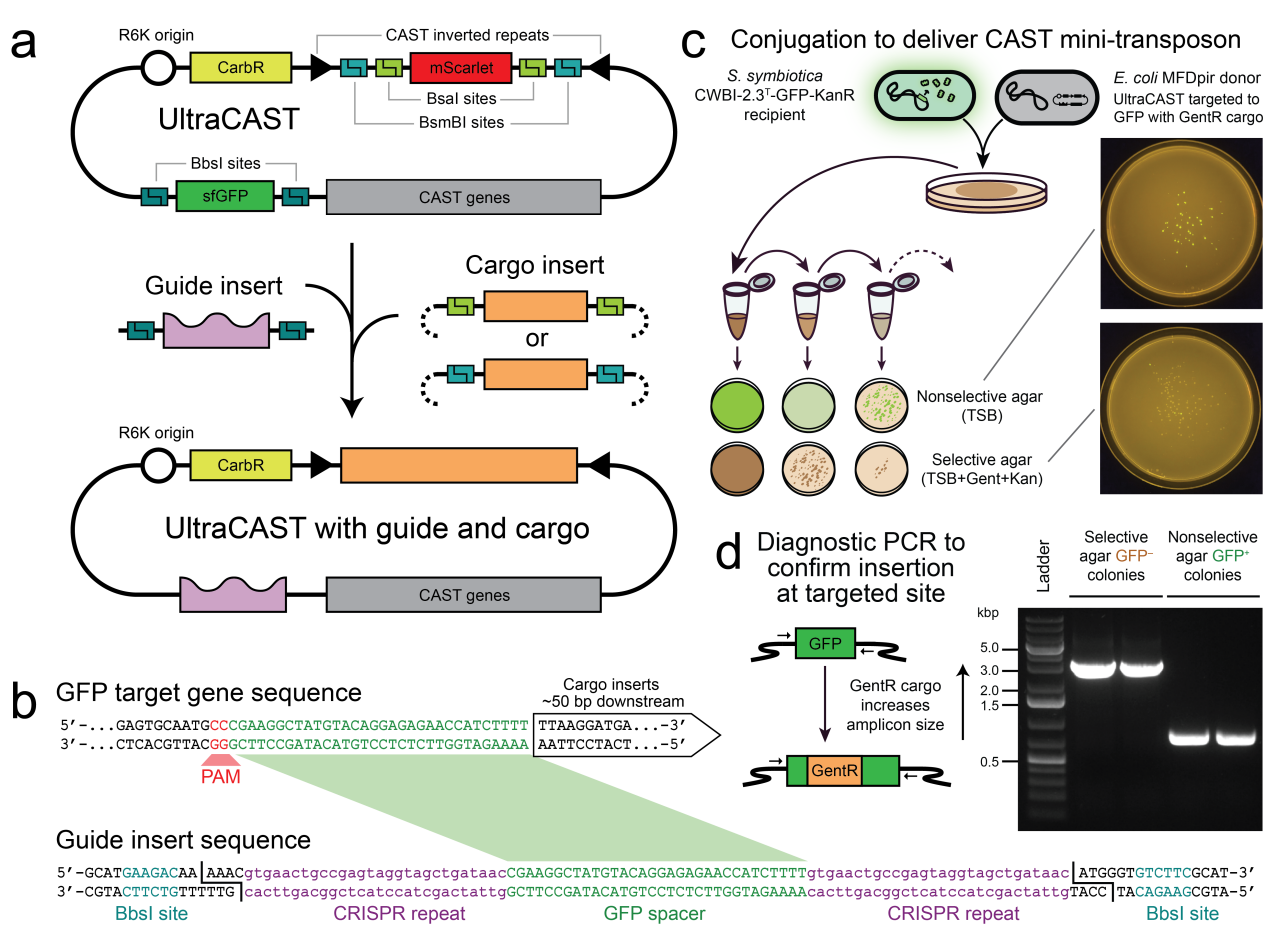
( **a** ) UltraCAST plasmid map and steps for cloning guide and cargo inserts. In the example, we replaced the sfGFP dropout with a guide sequence targeting GFP using BbsI Golden Gate assembly and replaced the mScarlet dropout with a gentamicin resistance cassette (GentR) using BsaI Golden Gate assembly. ( **b** ) Guide insert sequence design. Oligos are annealed to create the double-stranded piece of DNA encoding the guide RNA flanked with BbsI restriction sites. The sequence shown was designed for targeting the GFP gene integrated into the chromosome of *S. symbiotica* CWBI-2.3 ^T^ -GFP-KanR. ( **c** ) Delivery of the UltraCAST suicide plasmid from a donor *E. coli* strain to the recipient bacterium through conjugation followed by selection for insertion of the cargo cassette. In the example, loss of GFP expression from the *S. symbiotica* recipient in most cells on the selective plate indicates a high frequency of the on-target insertion event. Plates were visualized using a blue light transilluminator. ( **d** ) Diagnostic PCR to verify insertion at the targeted site. In the example, PCR amplicons with primers flanking the targeted GFP gene in the *S. symbiotica* chromosome show the expected increase in size after insertion in two different colony picks from the selective plate relative to the size in colonies with no insertion from a nonselective plate.

## Description

Metagenomic studies have discovered diverse bacterial communities in the environment and plant and animal microbiomes, but most of these bacteria have never been experimentally characterized. Even when they can be cultured, developing reliable genetic tools for non-model bacteria can be a daunting and time-consuming process of trial and error (Elston et al. 2023; Gilbert et al. 2023). Transposons are mobile genetic elements that are often among the first tools available for mutagenesis and genome editing of new microbial species. Most transposons either insert nonspecifically at many sites throughout a genome (e.g., Tn *5* ) or insert into one conserved site (e.g., Tn *7* ) (de Lorenzo et al. 1990; Craig 1991; Reznikoff 1993). To insert a DNA cargo or edit a specific site in a bacterial genome, homology-based recombination methods are typically used (Sharan et al. 2009; Lariviere et al. 2024), but these may require expressing phage recombinases or working out DNA delivery methods.

CRISPR-associated transposons (CASTs) are RNA-guided mobile elements related to Tn *7* that are widespread in bacteria (Peters et al. 2017; Rybarski et al. 2021; Klompe et al. 2022; Park et al. 2022; Faure et al. 2023). As with other transposons, they can be engineered into mini-transposons in which a cargo, located within the terminal inverted repeats of the transposon, can be mobilized by expressing transposon genes in *trans* (Strecker et al. 2019; Klompe et al. 2019; Vo et al. 2021; Klompe et al. 2022; Gelsinger et al. 2024). Refactored VcCASTs based on the type I-F system from *Vibrio cholerae* HE-45 have been shown to function in *Escherichia coli* and *Klebsiella, Pseudomonas* , and *Ralstonia* species (Klompe et al. 2019; Vo et al. 2021; Rubin et al. 2022; Gelsinger et al. 2024). VcCAST mini-transposons have >95% specificity for inserting their cargo at a site ~50 bp downstream of a match to their guide RNA sequence with a protospacer-adjacent motif (PAM) (Klompe et al. 2019; Vo et al. 2021). They may insert their cargo in either a forward or reverse orientation, with a higher preference for one orientation with certain guide RNAs and shorter cargos (Klompe et al. 2019). VcCAST mini-transposons have been used to insert >10-kb DNA cargos (Klompe et al. 2019), and they can likely mobilize even larger cargos, as the natural sizes of some type I-F CASTs surpass 80 kb (Klompe et al. 2022).

Here we present the UltraCAST, a VcCAST plasmid that improves on a previous design (Hu et al. 2024). The UltraCAST is a suicide vector with an R6K origin of replication that includes two Golden Gate compatible fluorescent protein dropout sequences for cloning in DNA inserts encoding a guide and cargo ( **Fig. 1a** ). A double-stranded DNA fragment encoding the guide RNA is cloned in using BbsI in place of the sfGFP dropout ( **Fig. 1b** ). The mScarlet dropout site can accept a cargo sequence containing either BsmBI or BsaI cut sites that generate overhangs compatible with either Stage 1 or Stage 2 assembly according to existing synthetic biology standards (Lee et al. 2015; Leonard et al. 2018). Guide and cargo cloning can be done in either order, facilitating use cases where a researcher wants to target different sites and/or species with the same cargo or when different cargos are to be inserted at the same site in one genome.

The assembled UltraCAST is delivered to the strain of interest through conjugation from a pir ^+^ *E. coli* donor strain. Since the R6K plasmid cannot replicate in recipient cells that are *pir ^–^* or otherwise incompatible with this origin of replication, CAST-mediated insertions of the mini-transposon cargo are selected in a single step. Unlike VcCAST configurations that rely on one or more replicative plasmids (Klompe et al. 2019; Vo et al. 2021; Gelsinger et al. 2024), this means that editing can take place in non-model bacterial strains for which no compatible plasmid origins are known and that there is no need to cure cells of the plasmid after editing is complete.

We initially verified that the refactored UltraCAST system functioned by using it to insert a chloramphenicol resistance cassette into the *lacZ* gene of *E. coli* REL606, the ancestor of a >35-year long-term evolution experiment (Lenski et al. 1991, Barrick et al. 2023). We have also used the UltraCAST to insert a gentamicin resistance cassette into various genes in *Serratia symbiotica* CWBI-2.3 ^T^ , a culturable gut symbiont of aphids that is related to strains that have evolved into vertically inherited intracellular endosymbionts (Sabri et al. 2011; Renoz et al. 2021). To date, we have used the UltraCAST to disrupt *aroG* , *esaR, esaI,* *leuB,* *sctN, cyaA, ridA,* and *pheA* in this species to study how these gene knockouts affect bacterial growth and aphid colonization.

This protocol describes how to use the UltraCAST to insert a DNA cargo at a specific position in the genome of a recipient bacterium. We demonstrate the steps and show expected results for inserting a ~2-kb gentamicin resistance cassette into the chromosome of *S. symbiotica* CWBI-2.3 ^T^ -GFP-KanR (Perreau et al. 2021). The on-target efficiency of insertion of the CAST mini-transposon into GFP in *S. symbiotica* was ~2.4% ( **Fig. 1c** ). A diagnostic PCR verified that the gentamicin resistance cassette was successfully integrated into the sfGFP gene ( **Fig. 1d** ). This protocol can be modified for other targets and cargos. This blueprint and the flexible UltraCAST plasmid should facilitate using this system to genetically engineer other non-model bacteria.

## Methods

**Design and clone a guide into the UltraCAST**

1. Design the DNA sequence encoding the CAST guide RNA by scanning the region being targeted for a 5ʹ-CC-3ʹ protospacer adjacent motif (PAM). Use the 32 bases following the PAM for the guide sequence. The CAST will insert ~50 bp downstream of the 3ʹ end of where the guide matches the genome. For the GFP example, we used 5ʹ-CGAAGGCTATGTACAGGAGAGAACCATCTTTT-3ʹ.

2. Append sequences that will be transcribed into the CRISPR array repeats (lowercase) and flanks needed for BbsI Golden Gate assembly (uppercase). Add 5ʹ-GCATGAAGACAAAAACgtgaactgccgagtaggtagctgataac-3ʹ before your guide sequence and 5ʹ-gtgaactgccgagtaggtagctgataacATGGGTGTCTTCGCAT-3ʹ after it. Order the full 120-base sequence and its reverse complement for DNA synthesis.

3. Resuspend oligos in nuclease-free water to 100 µM. Dilute and combine them in duplex buffer. We used a 10 µM final concentration of each oligo. Anneal the oligos to create a double-stranded DNA fragment by running this program in a thermocycler: 95°C (2 min), ramp to 12°C by 1°C per 30 s, 12°C (hold).

4. Clone the guide into the UltraCAST vector using BbsI Golden Gate assembly. We used 2.5 nM vector and 25 nM guide insert in a 20 µL reaction with 20 U NEB BbsI-HF enzyme and 800 U T4 DNA ligase in 1× rCutSmart buffer supplemented with 0.1 mM of ATP and ran this thermocycler program: 30 cycles alternating between 37°C (1 min) and 16°C (1 min), then final 60°C (5 min) and 12°C (hold) steps.

5. Transform the Golden Gate reaction into *E. coli* pir ^+^ cells and plate on LB agar with 100 µg/mL carbenicillin (LB-Carb agar) for selection. Incubate overnight at 37°C.

6. Pick one or more GFP negative colonies by inspecting plates on a blue light transilluminator. Grow liquid cultures in LB-Carb overnight at 37°C with shaking, miniprep plasmids, and verify that one has the intended guide by sequencing.

**Troubleshooting** : If there are no GFP negative colonies after transformation, try increasing the time at both 37°C and 16°C for each Golden Gate assembly cycle to 5 min and the number of cycles to 45.

**Clone a cargo into the UltraCAST**

1. The UltraCAST has a cargo site that accepts either a Type 2-4 YTK/BTK construct via BsaI Golden Gate assembly or a ConLS-ConRE construct via BsmBI Golden Gate assembly (Lee et al. 2015; Leonard et al. 2018). Miniprep part plasmids or create PCR products with compatible restriction sites and overhangs for cloning into this site.

2. Clone cargo into the UltraCAST using BsaI or BsmBI Golden Gate assembly. Use the same thermocycler conditions as for BbsI assembly to add the guide (see above). For the example, we performed BsaI assembly with 2.5 nM of UltraCAST vector and 25 nM of a Type 2-4 gentamicin resistance (GentR) cassette part plasmid in a 20 µL NEBridge (BsaI-HF v2) kit reaction.

3. Transform the Golden Gate reaction into *E. coli* pir ^+^ cells and plate on LB-Carb agar supplemented with additional antibiotic for a resistance cassette in the cargo, if applicable. We used 20 µg/mL gentamicin. Incubate overnight at 37°C.

4. Pick one or more mScarlet negative colonies by inspecting plates on a blue light transilluminator. Grow liquid cultures in LB-Carb overnight at 37°C with shaking, miniprep plasmids, and verify that one has the intended cargo by sequencing.

**Alternatives** : We created an equivalent Type 2-4 kanamycin resistance (KanR) cassette that can be used for organisms and cases where selection with this antibiotic is preferred. The guide and cargo inserts can be cloned in either order to facilitate making a family of plasmids with the same cargo and different guides or vice versa.

**Prepare the *E. coli* conjugation donor strain**

1. Transform the final UltraCAST plasmid with a guide and cargo into a pir ^+^ *E. coli* conjugation donor strain. Plate on LB-Carb plates supplemented with any additional nutrients needed for auxotrophic strains to grow. Incubate overnight at 37°C. We used *E. coli* MFDpir electrocompetent cells and cultured them with 0.3 mM 2,6-diaminopimelic acid (DAP).

2. Pick colonies and grow in at least 5 mL of LB with the same supplements overnight with shaking at 37°C.

**Pause Point:** These cultures can be frozen at –80°C with 15% w/w glycerol and later revived with overnight growth for conjugation.

**Alternative** : *E. coli* ST18 can be used as the pir ^+^ conjugation donor strain. In this case, supplement media with 50 μg/mL 5-aminolevulinic acid (ALA) instead of DAP.

**Conjugate into the recipient strain**

1. Grow a culture of the recipient strain. Time its growth so that a saturated culture or agar plate that can be scraped is available at the same time as a freshly grown overnight culture of the *E. coli* donor strain. We used *Serratia symbiotica* CWBI-2.3 ^T^ -GFP-KanR revived from a glycerol stock and grown in 5 mL TSB with shaking at 25°C for ~48 h.

2. Spin down donor and recipient cultures at 6800×g for 3 min. Wash each tube of cells three successive times with 1× PBS by removing supernatant and spinning down at 6800×g for 1 min. Resuspend the washed pellets in 1× PBS, diluting as necessary to reach an OD600 value of ~1 for each strain.

3. Mix together a total volume of 1 mL of washed donor and recipient cells. The ratio between the two strains should be adjusted so that it includes more of the slower-growing strain (usually the recipient). Spin down at 10,000×g for 1 min. Resuspend the combined pellet in 100 µL of 1× PBS. Plate the entire solution in a single drop on an agar plate on which both strains can grow. It should be supplemented with any nutrient the *E. coli* donor needs to grow and should not contain the antibiotic that will be used later to select for successful insertion of the cargo into the genome of the recipient. Once dry, incubate the plate under conditions where both strains can grow for long enough that there is some growth of the recipient. For the example, we used a 1:5 ratio of *E. coli* to *S. symbiotica* , plated on TSB-DAP, and incubated at 25°C overnight.

4. Scrape cells and resuspend in 1 mL of 1× PBS. Make a 10-fold dilution series in 1× PBS out to a 10 ^7^ dilution. Plate on selective and non-selective agar that lacks the nutrient needed by the auxotrophic donor. The selective agar should include the antibiotic for the resistance gene encoded in the CAST cargo. Incubate under growth conditions for the recipient strain. We used TSB-Gent-Kan agar and incubated at 25°C for 96 h. The gentamicin was to select for cargo insertion. The kanamycin was added because our *S. symbiotica* recipient strain already had this resistance marker in its genome.

5. Use diagnostic PCR reactions and/or genome resequencing to confirm insertion of the transposon cassette at the targeted site. In the example, successful knockouts were verified based on an increase in the size of a PCR amplicon spanning the insertion site and loss of GFP expression due to the mini-transposon cargo inserting into and inactivating this gene.

## Reagents

**Table:** 

**Reagent**	**Supplier/Catalog # or Recipe**
Dulbecco’s PBS	Sigma Aldrich (D8537)
*E. coli pir+*	Avantar TransforMax EC100D pir+ (75927-934)
*E. coli* MFDpir	Ferrières et al. 2010
*E. coli* ST18	German Collection of Microorganisms and Cell Cultures (DSM 22074)
Gentamicin Sulfate (Gent)	GoldBio (G-400-5)
Kanamycin Monosulfate (Kan)	GoldBio (K-120-5)
Carbenicillin (Disodium) (Carb)	GoldBio (C-103-5)
2,6-Diaminopimelic Acid (DAP)	Sigma Aldrich (D1377-5G)
5-Aminolevulinic Acid Hydrochloride (ALA)	Sigma Aldrich (A7793-1G)
UltraCAST Vector	Addgene (236111)
GentR Type 2-4 Part Plasmid	Addgene (236187)
KanR Type 2-4 Part Plasmid	Addgene (236186)
Guide RNA Oligonucleotides	Integrated DNA Technologies (Ultramer)
Duplex Buffer	100 mM Potassium Acetate, 30 mM HEPES, pH 7.5
Agar	Fisher Bioreagents (10153193)
Lysogeny Broth (LB)	For 1L: 10 g Tryptone, 5 g Yeast Extract, 10 g Sodium Chloride
LB Agar	For 1L: 10 g Tryptone, 5 g Yeast Extract, 10 g Sodium Chloride, 15 g Agar
Tryptic Soy Broth (TSB)	For 1L: 30 g of BD Bacto Tryptic Soy Broth (DF0370-17-3)
TSB Agar	For 1L: 30 g of BD Bacto Tryptic Soy Broth (DF0370-17-3), 15 g Agar
*S. symbiotica* CWBI-2.3 ^T^ -GFP-KanR	Perreau et al. 2021
ZymoPure Plasmid Midiprep Kit	ZymoPure II Plasmid Midiprep Kit (D4200)
BbsI-HF	New England Biolabs (R3539S)
NEBridge Golden Gate Assembly Kit (BsaI-HF v2)	New England Biolabs (E1601L)
NEBridge Golden Gate Assembly Kit (BsmBI-v2)	New England Biolabs (E1602L)
T4 DNA Ligase	New England Biolabs (M0202L)
rCutSmart Buffer	New England Biolabs (B6004S)
Adenosine 5'-Triphosphate (ATP)	New England Biolabs (P0756S)
